# Three-dimensional dynamics of fluctuations appearing during pellet ablation process around a pellet in a fusion plasma experiment

**DOI:** 10.1038/s41598-022-18239-z

**Published:** 2022-08-20

**Authors:** S. Ohshima, T. Suzuki, R. Matoike, G. Motojima, S. Kado, A. Mori, A. Miyashita, S. Kobayashi, T. Minami, A. Iwata, D. Qiu, C. Wang, M. Luo, P. Zhang, Y. Kondo, N. Nishino, T. Mizuuchi, H. Okada, S. Konoshima, S. Inagaki, K. Nagasaki

**Affiliations:** 1 Institute of Advanced Energy, Kyoto University, Kyoto, 611-0011 Japan; 2 Graduate School of Energy Science, Kyoto University, Kyoto, 611-0011 Japan; 3 National Institute for Fusion Science, Gifu, 509-5292 Japan; 4 SOKENDAI (The Graduate University for Advanced Studies), Gifu, 509-5292 Japan

**Keywords:** Plasma physics, Nuclear fusion and fission

## Abstract

Understanding pellet ablation physics is crucial to realizing efficient fueling into a high temperature plasma for the steady state operation of ITER and future fusion reactors. Here we report the first observation of the formation of fluctuation structures in the pellet plasmoid during the pellet ablation process by a fast camera in a medium-sized fusion device, Heliotron J. The fluctuation has a normalized fluctuation level of ~ 15% and propagates around the moving pellet across the magnetic field. By comparing the fluctuation structures with the shape of magnetic field lines calculated with the field line tracing code, we successfully reconstruct the spatio-temporal structure of the fluctuations during the pellet ablation process. The fluctuations are located at the locations displaced toroidally from the pellet and propagate in the cross-field direction around the pellet axis along the field line, indicating a three-dimensional behavior and structure of fluctuations. The fluctuation would be driven by a strong inhomogeneity formed around the pellet and invoke the relaxation of the gradient through a cross-field transport induced by the fluctuations, which could affect the pellet ablation and pellet fueling processes. Such fluctuations can be ubiquitously present at the inhomogeneity formed around a pellet in the pellet ablation process in fusion devices.

## Introduction

Efficient fueling to a high temperature plasma is a critical issue for the steady state operation of fusion reactors since only a small portion of neutral gas injected with a conventional gas puff fueling can penetrate across the separatrix and reach into the plasma core in high temperature plasmas^1,2^. The cryogenic pellet injection method is a promising tool to solve this issue, which injects a small hydrogen ice pellet into a high temperature plasma with fast speed and realizes an efficient fueling to the core plasma region compared to the conventional gas puff^3,4^. The pellet injection method is also applied for density profile control, suppression of instabilities, and diagnostic purposes^4^. The method has been technically developed well^5–7^, and a neutral gas and plasma shielding (NGPS) model has been developed including the description of the ionized pellet ablatant^8–12^. However, the experimental validation of the physics related to the pellet injection process has not yet been fully achieved due to the lack of ablation measurement, for example, regarding drift of plasmoid, plasmoid expansion, ablation process, particle deposition, plasma responses to pellet injection^4^. These processes are complicated, mediating interactions among different states of solid, gas, and plasma.

One of the difficulties in understanding the pellet ablation physics is that the measurement of the ablation is still challenging even nowadays because the plasmoid surrounding the ablation cloud has the strong inhomogeneity of plasma parameters within a small spatial extent and a three-dimensional shape elongated to the magnetic field lines, accompanied by fast dynamics (1–100 μs). Although the spectroscopic technique is a powerful tool to measure the pellet plasmoid parameters^13–15^, the spatial and temporal resolution of the measurement is not enough to understand the pellet ablation dynamics progressing with the fast time scale within a small spatial extent. The recent development of fast camera technology with higher time resolution has been expected to open a new horizon for understanding the dynamics of plasma^16–18^ including pellet physics. At present, a fast camera is an optimum tool to visualize the pellet dynamics and ablation process with high spatial and temporal resolutions^19–25^, although quantitative measurements of physical quantities are difficult.

In this study, we report the first observation of the fluctuation structures formed in the vicinity of a pellet ablation cloud on the fast camera measurement. The observed fluctuation propagates in the perpendicular direction to the magnetic field, which implies that this phenomenon with a three-dimensional feature could play an important role in the fueling process with the pellet ablation. The new finding gives us a novel insight into understanding the physics of the pellet ablation and fueling processes.

### Experimental set up

Heliotron J is a medium-sized helical device with the magnetic field strength B = 1.2 T on axis and the major/averaged minor radii of R/a = 1.2/0.15 m^26,27^. The magnetic field is generated with a single helical coil, two types of toroidal coils, and three sets of vertical field coils. The device is equipped with three heating methods of electron cyclotron heating (ECH), ion cyclotron resonance heating, and neutral beam injection(NBI) heating. The typical plasma parameters are the line averaged electron density of 1 × 10^18^–10^20^/m^3^, the electron temperature of Te < 3 keV, and the ion temperature of Ti < 400 eV.

A cryogenic pellet is injected into the Heliotron J plasma with the typical velocity of 260 ± 30 m/s with a pellet diameter of ~ 0.7 mm^28,29^. The pellet injection leads the plasma to the high density regime with higher stored energy in Heliotron J^29^.

**Figure 1:**
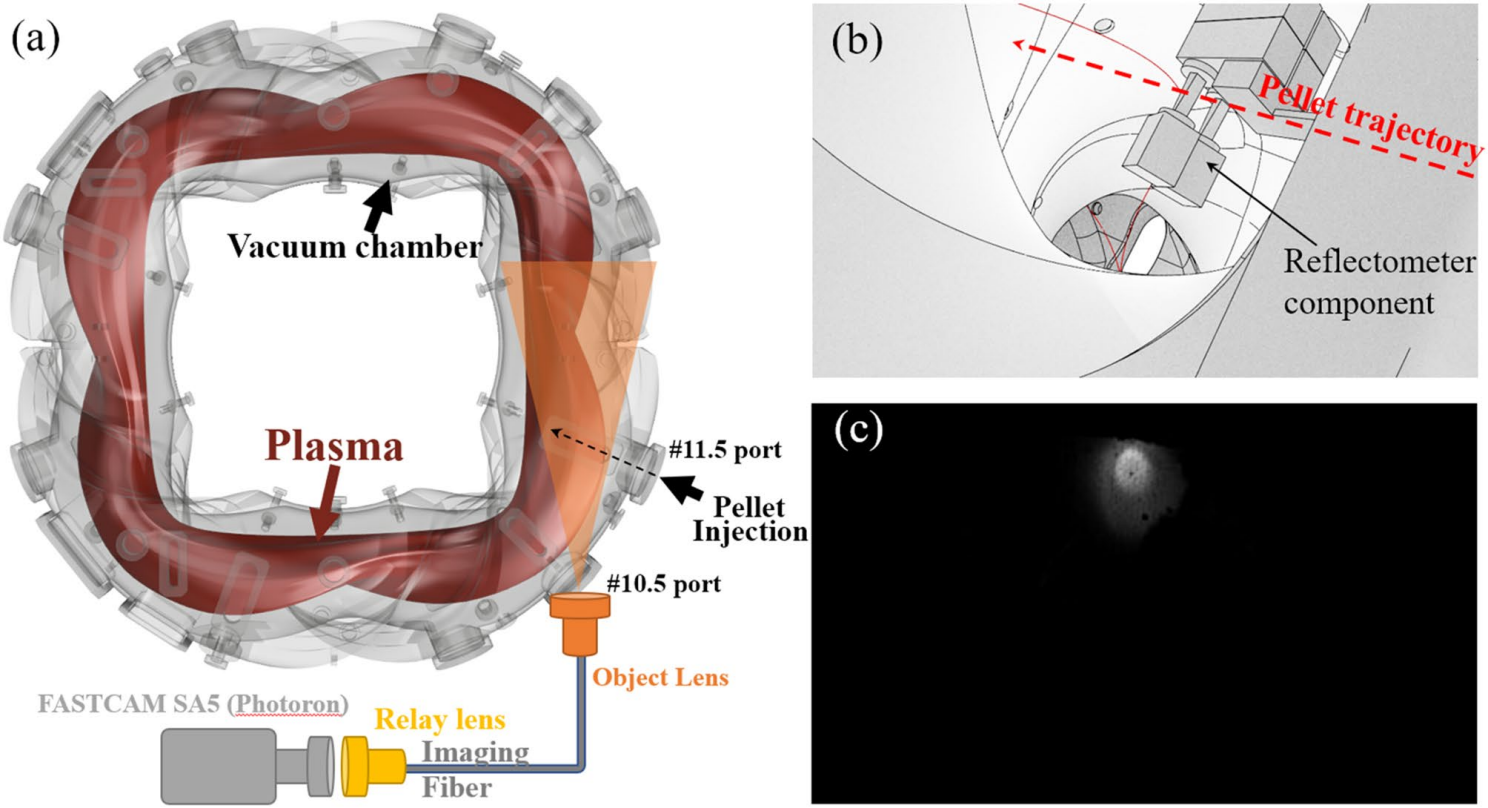
(**a**) Schematic of the fast camera system in Heliotron J. The figure was drawn by “Fusion 360”. (**b**) Field of view of the fast camera system reconstructed as a 3D model, drawn by “Blender”. (**c**) Typical snapshot image of the fast camera in a pellet experiment (shot#79188). See supplementary video 1.

Figure 1a shows a schematic of the fast camera system in Heliotron J. The camera system has a tangential view of the torus from the #10.5 port and observes the pellet trajectory, as shown in Fig. 1a and b, approximately from the perpendicular direction. The fast camera (FASTCAM SA5, Photron Co., Ltd) operates with a sampling rate of 100,000 fps, a spatial resolution of 320 × 192 pixels in this experiment^30,31^. Due to the influence of the strong magnetic field on the camera operation, the camera body is placed at a distance from the Heliotron J and the optical signal is transmitted by an imaging fiber.

The field of view of the camera system can be reconstructed in a three-dimensional model, as shown in Fig. 1b. Also, a typical snapshot of fast camera images and a movie are shown in Fig. 1c and as a supplementary video 1. By comparing the 3D model with the magnetic field lines calculated with a magnetic field tracing code and fast camera images, one can reconstruct the spatial structure of fluctuation observed around the pellet. The detail of the analysis and result is described in section “Experimental results and discussion”.

## Experimental results and discussion

### Observation of fluctuation structure during pellet ablation

In this experiment, a pellet was injected into the Heliotron J plasma with the line averaged electron density of 0.6 × 10^19^/m^3^. The target plasma was produced and sustained by 70 GHz ECH with the auxiliary heating of NBI. The injection power of ECH is ~ 250 kW, and the NBI power is ~ 150 kW.

**Figure 2:**
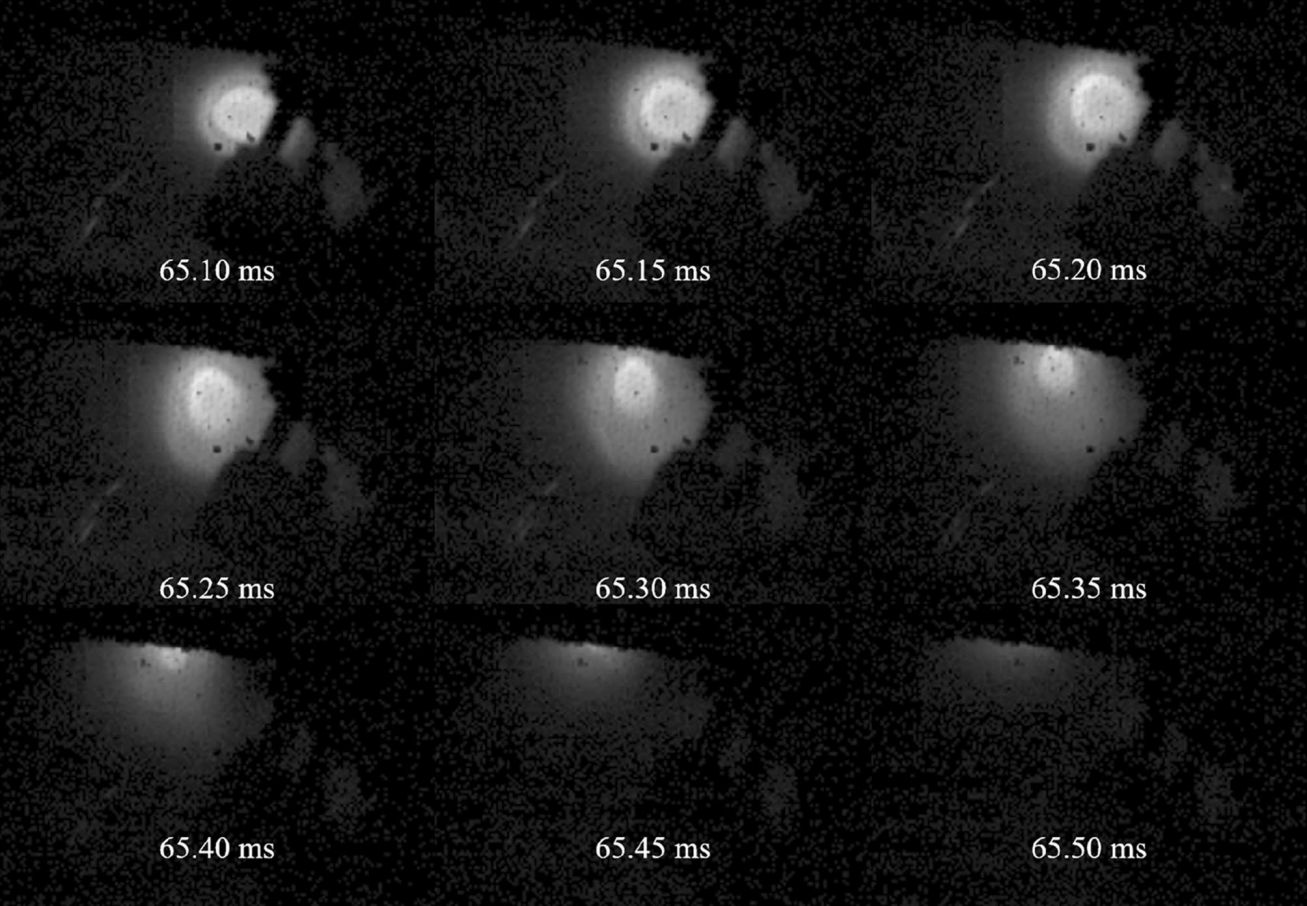
Sequential images of pellet injection in shot#79188. See supplementary video 2.

Sequential images of the pellet injection process taken by the fast camera are shown in Fig. 2, where typical snapshots are chosen and shown every 50 μs. The pellet velocity estimated from the images is around ~ 240 m/s, which is in good agreement with the design value of the pellet injection system^28^. In this experiment, fluctuation structure was observed inside the plasmoid in the vicinity of the ablation cloud, as can be seen in supplementary video 2. However, separation of the motions of the moving pellet and fluctuation structures is required to understand the fluctuation dynamics accurately.

**Figure 3:**
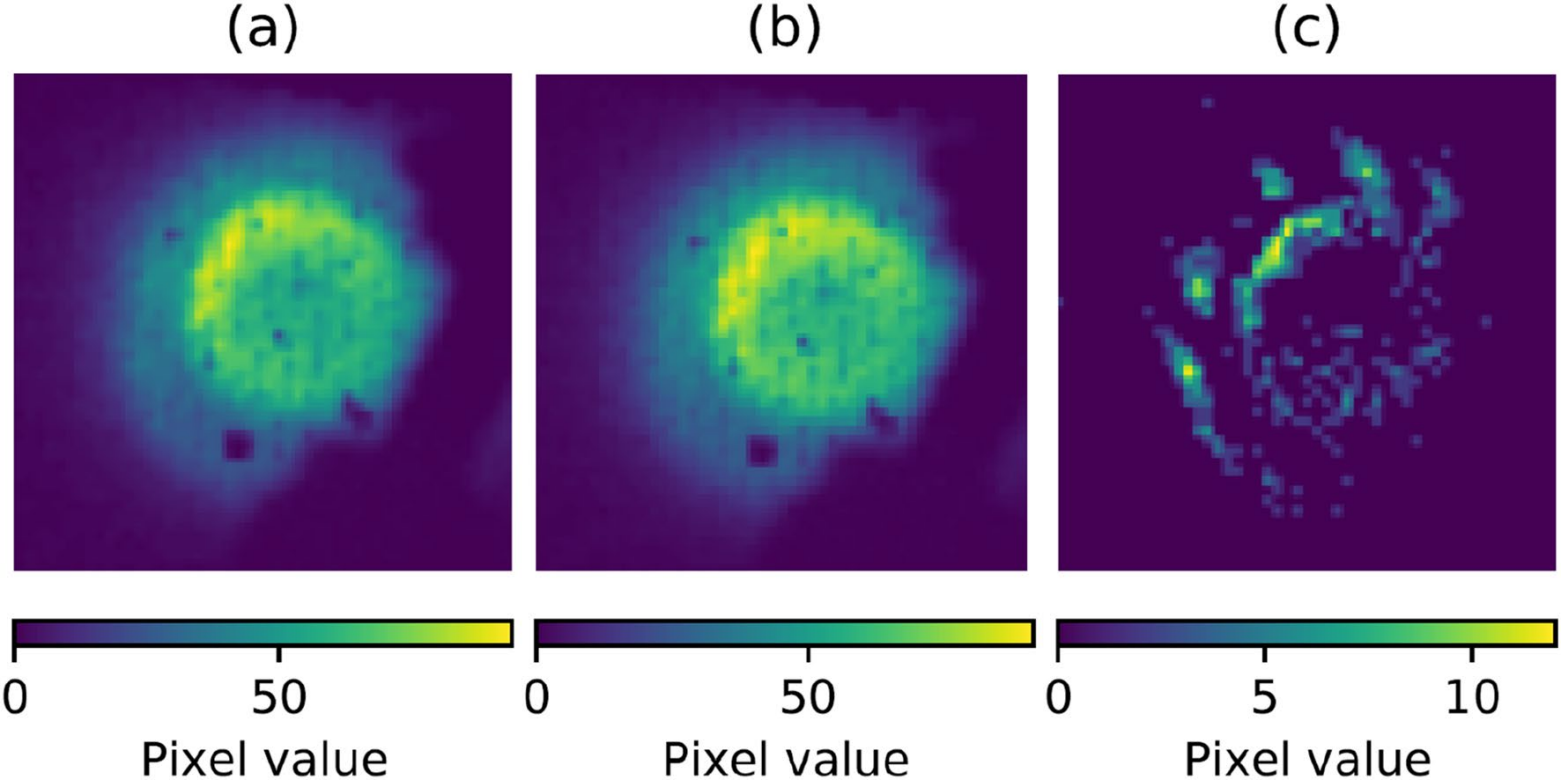
(**a**) Raw, (**b**) time averaging, and (**c**) fluctuation part component in shot#79188. See supplementary video 3.

To separate the pellet motion and the fluctuation dynamics around the pellet, the fluctuation part was extracted from an image on the pellet coordinates. The pellet coordinate is defined here as a relative coordinate to the center of gravity(CoG) of the light emission profile from the moving pellet, as follows. The CoG of the emission profile was evaluated as ***r***_***CoG***_ = *∫rI*(***r***)d*S/∫I*(***r***)d*S,* where *I*(***r***) is the light emission distribution from the pellet, ***r***_***CoG***_ is the coordinate of the CoG of the emission profile on each image, and *∫dS* represents a surface integral on each image. The fluctuation part is expressed as ∂*I*(***r-r***_***CoG***_) = *I*(***r-r***_***CoG***_)−< *I*(***r-r***_***CoG***_) > in the pellet coordinate, where the bracket term corresponds to the slowly varying part obtained with a moving averaging of the 50 μs time window. The raw, time-averaging and extracted fluctuation parts of pellet images are shown in Fig. 3**a**–**c** and supplementary video 3.

The observed fluctuation appears to originate from the pellet, not from the visualization of ambient turbulence structure accentuated by neutral gas originating from the pellet ablation. As is well known, turbulence is visualized with a fast camera by puffing a small amount of gas and highlighting the turbulence structure, which is called gas puff imaging^17,18,32–34^. A similar visualization of ambient turbulence in the background plasma would be possible during a pellet ablation process due to the neutral gas surrounding a pellet. However, the normalized density fluctuation level observed in this experiment is ~ 0.15 under a general assumption of *∂I/I* ~ ∂n_e_/n_e_. This fluctuation level is much higher than the turbulence fluctuation level in the core region of Heliotron J, which demonstrates the fluctuation is not identical to background turbulence. Note that turbulence fluctuation level is generally less than 1% in the core region in both tokamaks and stellarators^35^, and this situation is the same in Heliotron J^36^. It is therefore reasonable to conclude that the observed fluctuation is induced by the pellet itself.

From the extracted images in Fig. 3c and supplementary video 3, it is obvious that the observed fluctuations propagate around the pellet ablation cloud in the plasmoid. Due to the complexity of the three-dimensional magnetic field in Heliotron J, we then compare the fluctuation dynamics with the magnetic field structure and discuss the spatio-temporal structure of fluctuations in the following section.

### Three-dimensional dynamics of observed fluctuations

**Figure 4:**
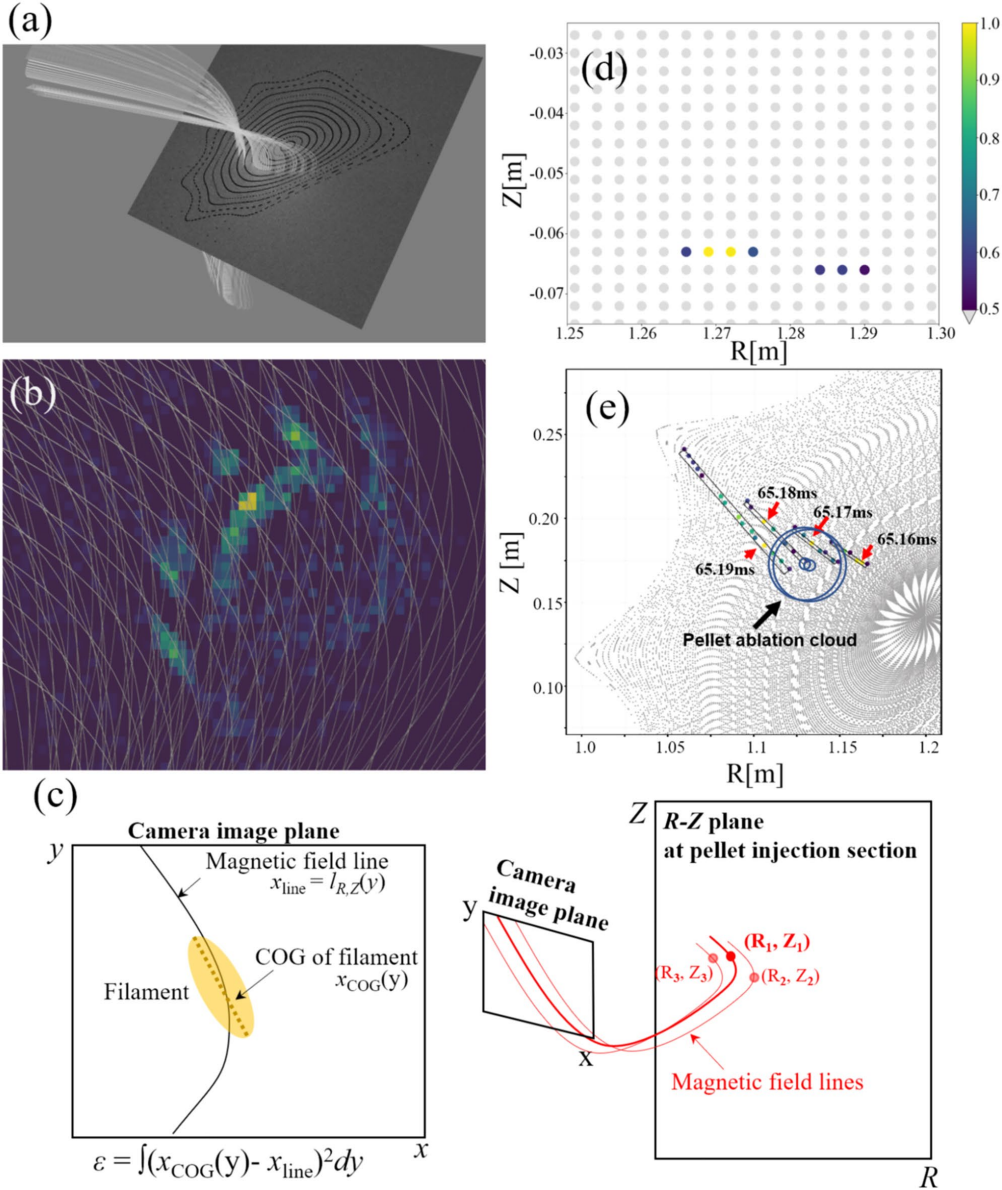
(**a**) Flux surface at the pellet injection toroidal section (#11.5) and representative magnetic field lines observed from the camera on the 3D model drawn by “Blender”. (**b**) Superimposed image of fluctuation structure and typical magnetic field lines. Different magnetic field lines are stacked with different tilt angles in the depth direction of the camera’s line of sight. (**c**) A schematic of the analysis procedure for localizing fluctuation. (**d**) A typical distribution of scoring parameter *s,* projected on the *R-Z* plane from *x–y* coordinates, at the toroidal section of the pellet injection port with a 3 mm spatial resolution. Only the points with s > 0.5 are shown as color here. (**e**) Time evolution of parameter *s* for a single, identical fluctuation. The color of points represents the *s* value, as well as (**d**). The circles indicate the ablation cloud of the pellet. While the fluctuation propagates around the pellet, the pellet itself does not move so much compared with the motion of the fluctuation structure. Note that the field line location where the fluctuation exists can be projected on the poloidal cross-section of the pellet injection port, as shown in (**c**). The detail of the three-dimensional motion of fluctuation is discussed in Figs. 5 and 6.

By comparing the shape of the fluctuations with the magnetic field lines, the position of each fluctuation can be identified, and thereby the spatio-temporal structure of each fluctuation can be reconstructed. The magnetic surface at the toroidal section of the pellet injection port and typical magnetic field lines are shown in Fig. 4a. Here the magnetic field structure is calculated with the field tracing code, KMAG^26,37^. Since magnetic field lines with different tilt angles are stacked in the depth direction of the camera's line of sight, as exhibited in Fig. 4b, a degree of coincidence between magnetic field lines and fluctuation structures is evaluated to identify the fluctuation position.

**Figure 5:**
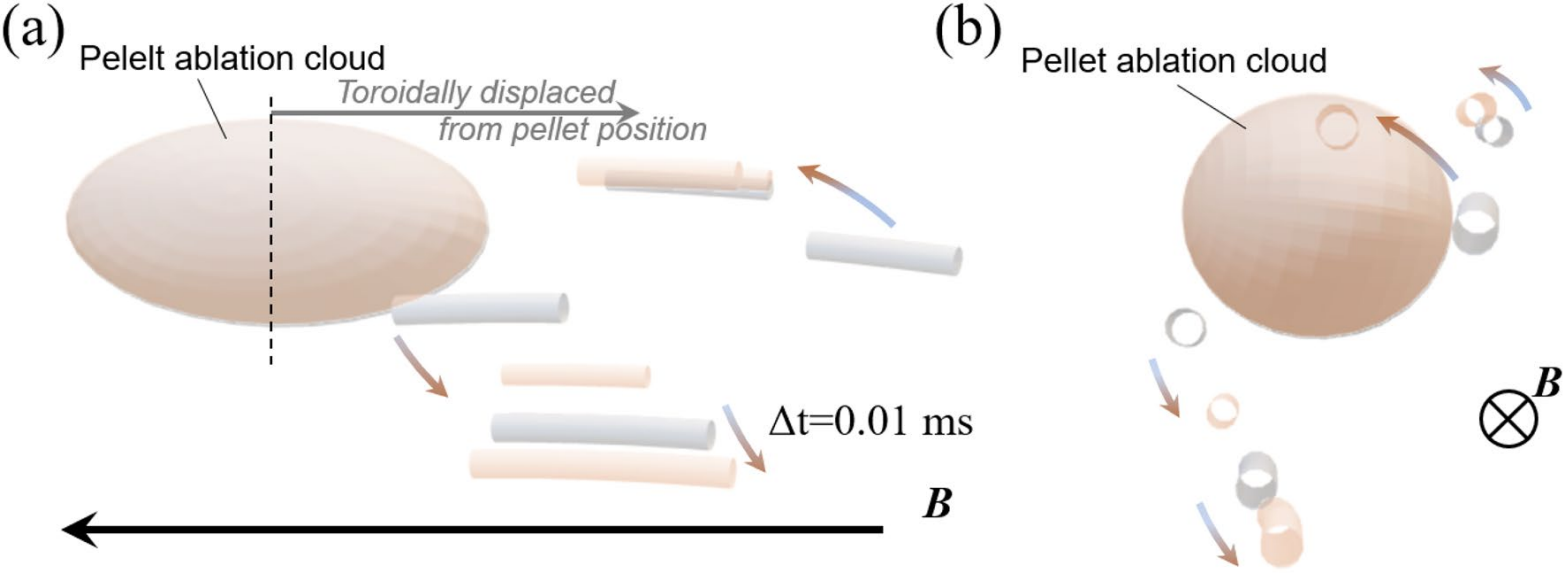
(**a**) Reconstructed fluctuation structure on the 3D model surrounding the pellet ablation cloud from the line of sight approximately in the perpendicular and (**b**) parallel directions to the magnetic field. Different color indicates fluctuation structure at different timing with the time interval of 0.01 ms. Fluctuations are located at the positions toroidally displaced from the pellet and rotate across the magnetic field around the pellet axis. The figures were drawn by “Blender”.

**Figure 6:**
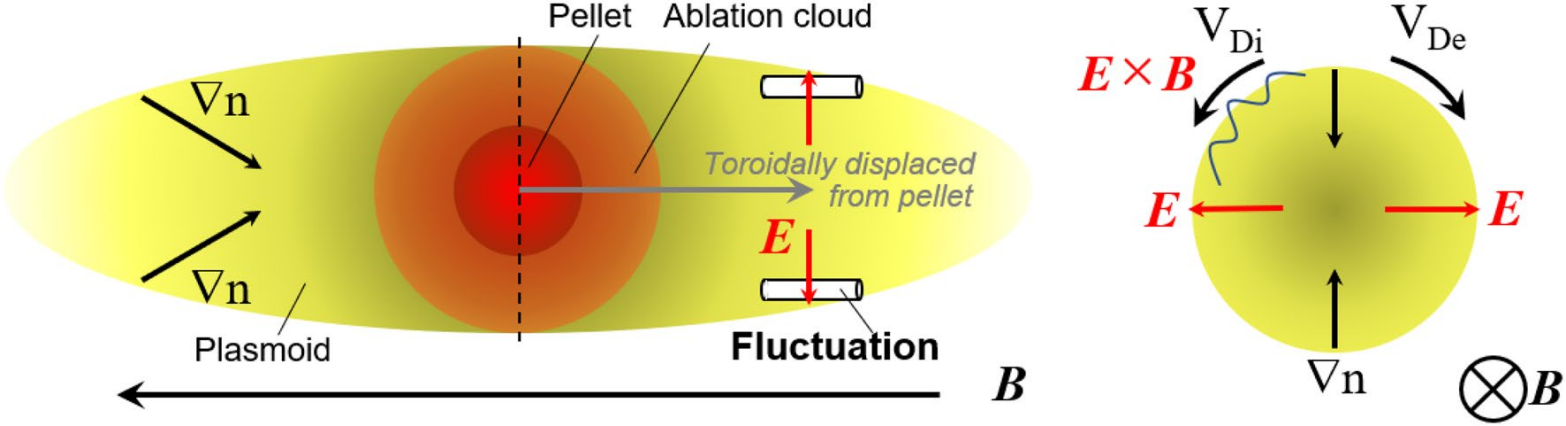
Schematic of fluctuation driving mechanism. For simplicity, the polarization electric field is not shown in this figure.

In this analysis, one can consider plasma to be transparent, and opacity is not an issue, based on the following consideration. The electron density of the pellet plasmoid close to the ablation cloud is about 10^21^/m^3^, estimated from the Stark broadening of Hα measured with the visible spectrometer in a typical pellet experiment on Heliotron J. At the density, optical thickness could need to be considered. It should be noted that, however, the spectrometer is not a spatially resolved measurement, and the estimated value corresponds to the density at the brightest Hα region, which is the ablation cloud (or pellet plasmoid) closest to the pellet core, as shown in Fig. 3a. The observed fluctuations are located at a distance from the pellet core region, and therefore the opacity does not need to be taken into consideration in this analysis.

A schematic of the analysis procedure for localizing fluctuation is shown in Fig. 4c. Based on the least-squares method, the magnetic field line matching a certain fluctuation structure is searched to evaluate a parameter *ε* defined as *ε* = *∫*(*x*_COG_(y)−*x*_line_)^2^*dy*, as shown on the left side of Fig. 4c. Here *x*_*COG*_*(y)* = *∫∂I(x,y)· xdx/∫∂I(x,y)dx* is the CoG of the emission profile in the *x* direction at an arbitrary *y* position, *∂I*(*x,y*) is the emission profile of the fluctuation part on the *x–y* plane of each image, and *x*_line_ represents the coordinate of a magnetic field line expressed as *x*_*line*_ = *l*_*R,Z*_*(y)*, projected on the *x–y* plane of each image. The *ε* values are evaluated for all the magnetic field lines which intersect with the fluctuation on each image, and then at the minimum *ε* value, *ε*_*min*_, one can find the magnetic field line on which the fluctuation likely localizes.

For simplicity of interpretation, by normalizing and inverting *ε*, we introduce a scoring parameter expressed as *s(R, Z)* = *ε*_*min*_*/ε(R, Z)*. The fluctuation with the filament structure has the most likelihood to be located on the field line where *s* has the maximum value of 1(*ε* has the minimum value *ε*_*min*_). Since each magnetic field line on the camera’s view intersects at each point on the *R–Z *plane as shown on the right side of Fig. 4c, the *s* and *ε* parameters can be plotted on the *R–Z* coordinates at the toroidal section of the pellet injection port(at #11.5 toroidal section). Here the full width at half maximum for the *s* value on the *R–Z* plane is regarded as an error, in other words, spatial extent in the range of 0.5 < *s* < 1 corresponds to the uncertainty of fluctuation localization.

A typical result of scoring parameter *s* distribution for a single fluctuation structure is plotted on the *R–Z* plane in Fig. 4d, with a 3 mm spatial resolution. The *s* value is plotted as color, and the plots with *s* < 0.5 are shown as grey. In this case, the fluctuation should localize on the magnetic field line passing through the coordinate of (*R, Z*) = (1.27, − 0.063) at the toroidal section of the pellet injection port. As shown in the figure, good localization of the fluctuation is possible at ~ 6 mm in the *Z* direction corresponding to the vertical direction of a camera image, whereas there exists considerable uncertainty in the *R* direction, of ~ 25 mm, in the depth direction of the camera’s sightline.

The large error in the *R* direction could be attributed to the specific magnetic field configuration of Heliotron J and the line-integral effect in the camera's depth direction. Although a larger variation in the tilt angle of magnetic field lines in the camera-depth direction is favorable for identifying the fluctuation position in the R-direction, the tilting of the magnetic field lines does not vary much in the depth direction due to the small magnetic shear in the Heliotron J configuration. Moreover, due to the line-integral effect arising from the transparency of the plasma, the filament axis (filament CoG) on the camera image would become thicker and less sharp, which is also unfavorable for localizing fluctuation. These two factors would contribute to the increase in error in the R direction, although it is difficult to evaluate these effects independently.

Based on the method described above, we extract the spatio-temporal behavior of each fluctuation. As an example, the time evolution of parameter *s* for a single fluctuation structure is plotted in Fig. 4e on the *R–Z* plane with the flux surfaces at the pellet injection port (indicated in Fig. 1a). The color of the points represents the *s* value, as well as Fig. 4d. The circles indicate the position of the pellet ablation cloud. As is seen from the figure, the fluctuation position rotates in the anti-clockwise direction around the ablation cloud, while the pellet position does not move so much. This result demonstrates that the observed fluctuation propagates in the cross-field direction around the moving pellet across a flux surface but is not localized at a certain flux surface, unlike usual turbulence.

In the same way, we reconstructed the spatio-temporal behavior of fluctuation structures surrounding the pellet ablation cloud, as shown as schematic in Fig. 5a and b. Different colors indicate the reconstructed structures at different timing with a time lag of *Δt* = 10 μs. The location of fluctuations is displaced toroidally from the pellet. As the pellet moves in plasma, each fluctuation propagates in the ion diamagnetic direction around the moving pellet with the speed of 1000–3000 m/s, which is significantly faster than the pellet motion (~ 240 m/s) itself. The dynamics of fluctuations evoked by the pellet exhibit a three-dimensional feature.

The driving and propagation mechanisms of the observed fluctuation are critical issues for understanding pellet physics. One can immediately notice that one-dimensional models cannot describe such a three-dimensional structure and motion of the fluctuation. One interpretation is that a strong density gradient inside the pellet plasmoid across the magnetic field can be a source of the fluctuation, i.e., drift instability observed universally in plasmas. The fluctuation propagation direction is in the ion diamagnetic direction, and this is the opposite direction of drift instabilities driven by electron density gradient, however, an electric field could account for the determination of fluctuation propagation, rather than the drift velocity, as observed in various plasma experiments^38,39^.

Other possible interpretations for the driving mechanism include the followings. The observed fluctuation might also be related to the “striation” caused by the *m* = 1 Rayleigh–Taylor instability^40^, as observed in the ablation process in the pellet experiments^15,22,25,41,42^. Although only the m = 1 mode has attracted attention so far as a mechanism of striation^40^, if the instability with a higher *m* mode number is destabilized, the instability should resemble the fluctuations observed in this experiment. In addition, an MHD simulation study suggests that magnetic field perturbation can be induced by plasmoid^43^. More systematic experiments in the wider parameter range are required to identify the driving mechanism to compare the experimental results and theories mentioned above for the basic properties of fluctuation, such as frequency, wavenumber, and propagation speed.

If one assumes the electric field dominantly determines the destabilized fluctuation propagation, the velocity of ***E*** × ***B*** drift of ~ 1000–3000 m/s should be induced by the perpendicular electric field of ~ 10–30 V/cm at the magnetic strength *B* = 1 T. An electric field in the perpendicular direction to a field line can be present even in the plasmoid^44^, although some modeling studies neglect the electric field inside a plasmoid along a field line since the potential structure is homogenized along a field line quickly due to the high conductivity inside the plasmoid^45^. A schematic of this simple interpretation described above is summarized in Fig. 6. A strong inhomogeneity formed around the pellet can induce such perturbation of the electric field perpendicular to the magnetic field. It should be noted that negative Er is predicted from the reference^40^, however, energetic ions produced with the 24 kV NBI could affect the electric field distribution inside the plasmoid, which might make the simple picture of the reference more complex. Controlling the power of NBI may vary the velocity and could change the direction of fluctuation rotation, which is beyond the scope of present work but will be an interesting subject for future work.

Regardless of instability type, a destabilized fluctuation generally causes a relaxation of the gradient of plasma parameters. The fluctuation appearing in the ablation process is predicted to induce transport that causes a relaxation of the gradient in the cross-field direction, thereby affecting fueling by a pellet. Such fluctuations could universally exist during the pellet ablation process inside the pellet plasmoid, contributing to the pellet ablation and fueling process. The observation, however, is expected to be difficult without a fast camera with higher spatial and temporal resolutions, because such fluctuations would appear as a small-scale instability driven by strong inhomogeneity within a small spatial extent inside the pellet plasmoid.

In this experiment, the fluctuations were observed only in the pellet traveling direction on the front side of the pellet within the camera's field of view. If our simple hypothesis described in Fig. 6 is valid, the fluctuation structure would exist on the rear-side of the traveling direction and behind the pellet, although toroidal asymmetry of fluctuation structure could exist, resulting from the asymmetry in ion velocity distribution function introduced by NBI^46^. The spatio-temporal structures of fluctuation should link with the particle deposition in the pellet fueling process, and systematic experiments in different heating schemes and different magnetic field configurations, for example, with a reversed field are necessary.

Finally, it should be mentioned that, in this experiment, the plasma density was lower than in the standard pellet experiment, resulting in moderate ablation of the pellet. This situation coincidentally facilitated the observation of fluctuations inside the plasmoid. Even in the standard conditions for pellet experiment with higher density, similar fluctuations can be observed in Heliotron J, however, it is difficult to clearly visualize the spatial structure of fluctuations using the present fast camera because the emission profile from the pellet is smaller than in this experiment, and the emission fluctuates more strongly and faster, probably due to the stronger gradient in the more intense pellet ablation. Further study with a fast camera having higher temporal and spatial resolutions is necessary for a more general understanding of the fluctuation inside the pellet plasmoid.

## Conclusions

The formation and propagation of fluctuation structures in the vicinity of the pellet ablation cloud have been visualized and discussed through the analyses of the fast camera images in Heliotron J. The normalized fluctuation level is 15% and is significantly higher than the turbulence level in background turbulence, which indicates that the pellet invoked the fluctuation. The spatiotemporal structure of the fluctuations in the pellet plasmoid was reconstructed by comparing fluctuation structures with the magnetic field line calculated with the field line tracing code KMAG. The fluctuations are present at the location displaced from the pellet in the toroidal direction and propagate in the cross-field direction, which implies that the fluctuation-induced cross-transport would affect the pellet ablation/fueling process. The three-dimensional dynamics of fluctuations observed in this experiment cannot be described by a one-dimensional model. The fluctuations driven by a strong inhomogeneity of plasma parameters around a pellet could exist ubiquitously during the pellet ablation, which would bring a modification of the present understanding of pellet ablation physics. Further experimental studies dedicated to observing a pellet ablation with a higher spatial and temporal resolution are required to understand the behavior of fluctuation under different experimental conditions, such as different heating schemes and magnetic configurations with a wider plasma parameter range.

## Supplementary Information

Supplementary Video 1.

Supplementary Video 2.

Supplementary Video 3.

## Data Availability

The data that support the findings of this study are available upon reasonable request from the corresponding author (S.O).

## References

[1] Baylor LR (2007). Pellet fuelling and control of burning plasmas in ITER. Nucl. Fusion.

[2] Combs SK, Baylor LR, Meitner SJ, Caughman JBO, Rasmussen DA, Maruyama S (2012). Overview of recent developments in pellet injection for ITER. Fusion Eng. Des..

[3] Milora SL (1981). Rview of pellet fueling. J. Fusion Energy.

[4] Pégourié B (2007). Review: Pellet injection experiments and modelling. Plasma Phys. Control. Fusion.

[5] Milora SL (1989). Review of hydrogen pellet injection technology for plasma fueling applications. J. Vac. Sci. Technol..

[6] Combs SK (1993). Pellet injection technology. Rev. Sci. Instrum..

[7] Verma SK (2020). A review of pellet injector technology: Brief history and recent key developments. Fusion Sci. Technol..

[8] Pegourie B, Picchiottino JM, Drawin HW, Geraud A, Chatelier M (1993). Pellet ablation studies on TORE SUPRA. Nucl. Fusion.

[9] Baylor LR (1997). An international pellet ablation database. Nucl. Fusion.

[10] Garzotti L, Pégourié B, Géraud A, Frigione D, Baylor LR (1997). Neutral gas and plasma shielding scaling law for pellet ablation in maxwellian plasmas. Nucl. Fusion.

[11] Rozhansky VA, Senichenkov IY (2005). On the ablation models of fuel pellets. Plasma Phys. Rep..

[12] Pégourié B (2005). Modelling of pellet ablation in additionally heated plasmas. Plasma Phys. Control. Fusion.

[13] Motojima, G., Sakamoto, R., Goto, M., Yamada, H. & LHD experiment group. Spectroscopic diagnostics for spatial density distribution of plasmoid by pellet injection in the large helical device. *Plasma Fusion Res.* **5**, S1033–S1033 10.1585/pfr.5.s1033 (2010).

[14] McNeill DH, Greene GJ, Newburger JD, Owens DK (1991). Spectroscopic measurements of the parameters of the ablation clouds of deuterium pellets injected into tokamaks. Phys. Fluids B.

[15] Müller HW (2002). High β plasmoid formation, drift and striations during pellet ablation in ASDEX upgrade. Nucl. Fusion.

[16] Kirk A (2006). Filament structures at the plasma edge on MAST. Plasma Phys. Control. Fusion.

[17] Maqueda RJ (2003). Gas puff imaging of edge turbulence (invited). Rev. Sci. Instrum..

[18] Zweben SJ (2009). GPI measurements of edge and SOL turbulence across the L-H transition in NSTX. Transition.

[19] Mishra JS, SaKoćamoto R, Matsuyama A, Motojima G, Yamada H (2011). Observation of three-dimensional motion of the pellet ablatant in the Large Helical Device. Nucl. Fusion.

[20] Bortolon A (2019). Ablation of solid pellets induced by supra-thermal ions in the far scrape-off layer of DIII-D plasmas. Nucl. Fusion.

[21] Sakamoto R, Yamada H (2011). Observation of cross-field transport of pellet plasmoid in LHD. Plasma Fusion Res..

[22] Sakamoto, R., Masuzaki, S., Miyazawa, J. & LHD Experimental Group. Observation of striation in collapsing plasma. *Plasma Fusion Res.* **1**, 019; 10.1585/pfr.1.019 (2006).

[23] Sakamoto R (2004). Observation of pellet ablation behaviour on the large helical device. Nucl. Fusion.

[24] Garzotti L (2010). Observation and analysis of pellet material δb drift on MAST. Nucl. Fusion.

[25] Panadero N (2018). Experimental studies and simulations of hydrogen pellet ablation in the stellarator TJ-II. Nucl. Fusion.

[26] Wakatani M (2000). Study of a helical axis heliotron. Nucl. Fusion.

[27] Obiki T (2001). First plasmas in Heliotron J. Nucl. Fusion.

[28] Motojima G (2016). Injection barrel with a tapered structure for a low speed and small size cryogenic hydrogen pellet in medium-sized plasma fusion devices. Rev. Sci. Inst..

[29] Motojima G (2019). High-density experiments with hydrogen ice pellet injection and analysis of pellet penetration depth in Heliotron J. Plasma Phys. Control. Fusion.

[30] Nishino N (2011). Peripheral plasma measurement during SMBI in Heliotron J using fast cameras. J. Nucl. Mater..

[31] Nishino N (2019). Estimation of three-dimensional structure on peripheral fluctuation using fast camera images and magnetic field calculation in Heliotron J. Nucl. Mater. Energy.

[32] Zweben SJ (2007). Edge turbulence measurements in toroidal fusion devices. Plasma Phys. Control. Fusion.

[33] Shesterikov I (2012). Direct evidence of eddy breaking and tilting by edge sheared flows observed in the TEXTOR tokamak. Nucl. Fusion.

[34] Zweben SJ (2010). Quiet periods in edge turbulence preceding the L-H transition in the National Spherical Torus Experiment. Phys. Plasmas.

[35] Liewer PC (1985). Measurements of microturbulence in tokamaks and comparisons with theories of turbulence and anomalous transport. Nucl. Fusion.

[36] Kobayashi S (2016). Development of beam emission spectroscopy for turbulence transport study in Heliotron J. Rev. Sci. Instrum..

[37] Nakamura Y (1993). Low-n mode stability analysis for l-2 Hliotron/Torsatron by VMEC-STEP Code. Plasma Fusion Res..

[38] Conway GD, Schirmer J, Klenge S, Suttrop W, Holzhauer E (2004). Plasma rotation profile measurements using Doppler reflectometry. Plasma Phys. Control. Fusion.

[39] Hirsch M, Holzhauer E, Baldzuhn J, Kurzan B, Scott B (2001). Doppler reflectometry for the investigation of propagating density perturbations. Plasma Phys. Control. Fusion.

[40] Parks PB (1996). Theory of pellet cloud oscillation striations. Plasma Phys. Control. Fusion.

[41] De Kloe J, Noordermeer E, Lopes-Cardozo NJ, Oomens AAM (1999). Fast backward drift of pellet ablatant in tokamak plasmas. Phys. Rev. Lett..

[42] Rozhansky V, Veselova I, Voskoboynikov S (1995). Evolution and stratification of a plasma cloud surrounding a pellet. Plasma Phys. Control. Fusion.

[43] Ishizaki R, Nakajima N (2011). Magnetohydrodynamic simulation on pellet plasmoid in torus plasmas. Plasma Phys. Control. Fusion.

[44] Parks PB, Lu T, Samulyak R (2009). Charging and E×B rotation of ablation clouds surrounding refueling pellets in hot fusion plasmas. Phys. Plasmas.

[45] Runov A, Aleynikov P, Arnold AM, Breizman BN, Helander P (2021). Modelling of parallel dynamics of a pellet-produced plasmoid. J. Plasma Phys..

[46] Matsuyama A, Pégourié B, Sakamoto R, Mishra JS, Motojima G, Yamada H (2012). Over-ablation and deflection of hydrogen pellets injected into neutral beam injection heated plasmas in the Large Helical Device. Plasma Phys. Control. Fusion.

